# Monthly Summary

**Published:** 1893-05

**Authors:** 


### Monthly Summary.

THE DENTISTS AT A DISADVANTAGE.—Although modern skill and science have not yet been able to abolish death, they can do much to conceal the ravages of time and the advance of age. False teeth, false eyes, false hair and false legs and arms are now fashioned to imitate nature's handiwork so cunningly that old men and woman may be made to look almost as good as new, whether they feel so or not. But if all the artists and professional gentlemen who minister after this fashion to the needs of the human form divine shared the views of Dentist Charles A. Van Duzee, of Minneapolis, some of those who are "made up" by the help of these cunning artificers might have occasion to tremble for their borrowed charms. Dr. Van Duzee it appears, made a set of teeth for a citizen of Minneapolis, and when he failed to pay for them got a judgement against the delinquent and endeavored to seize the teeth on execution. The defendant refused to give up the teeth, which were firmly fixed in his mouth, and the sheriff not desiring to violate the personal rights of a citizen, or to have his fingers bitten in the attempt to secure the property, asked the court for further instructions. The dentist's attorney argued that the court should order the defendant to surrender the teeth, and if he did not comply, have him arrested for contempt. The defendant contended that a man's person, though but a tenement of clay, was his absolute property for life, and that the teeth being on his premises, the sheriff would be a trespasser if he undertook to insert his fingers into defendant's mouth to remove them. He claimed further that the teeth had become part of his person, and that the court had no more right to drag them out of his head than it had to tear off one of his limbs. In his opinion the court, Judge Kelly, said:

"It is suggested these teeth are a part of defendants anatomy. It does not appear how securely they are attached, but the defendant evidently entertains great attachment for them. They have become so to speak, fixtures. And whether or not they may be removed without serious injury to the freehold does not clearly appear. The court cannot take judical notice of the manner false teeth are kept in place. Though not attached to the reality in the ordinary sense, yet practically speaking they are, for all flesh is dust. Suitable food is necessary to good health. Nature (and art in this case) provides teeth for mastication. It will not do to say that defendant may subsist comfortably on soups and hash. The one as a steady diet, is thin and unsatisfactory; the other, always mysterious. Conceding the plaintiff holds a judgment against this defendant for an unpaid portion of the purchase price of these teeth, and defendant has no other property subject to execution, and that goods sold and delivered are not, under our law, exempt from execution and sale for the purchase price, these teeth having become a part and parcel of defendant's anatomy. are while so used and worn, not subject to levy and sale. Plaintiff's contention leads to queer results. If I applly the law as plaintiff insists where will the court draw the line? Legs, arms, eyes, wigs, curls and bangs—the false and artificial of course—may all be brought to light and within the dread power of the sheriff."—*The Baltimore Sun.*

**DIFFUSE CELLULITIS OF THE NECK DUE TO CARIOUS TEETH.**—An interesting case of Diffuse Cellulitis of the neck, apparently of dental origin, recently occurred at St. Mary's hospital. The patient, when examined, was found to have two or three carious teeth in the lower jaw, one of which especially had given him trouble for about a month, the cheek being swoolen on the same side. Four days before he was admitted he had been seen by a doctor and advised to "come in" that same day, as his neck was then much swoolen and he had difficulty in swallowing, and his voice was husky. On the day of going into the hospital he could only speak in a whisper, had been unable to swallow any solid food for about four days, was very weak and had difficulty in breathing. His neck was greatly swoolen all round, especially on the right side, where the carious tooth was. His temperature was 161.8. He complained of considerable pain, and could scarcely open his mouth at all. Ether was administered, during which he expired. The *post-mortem* showed that there were signs of old chronic periostitis at the root of one of the lower molars on right side, and an abscess containing about ℥ i. of pus extending down from the jaw into the cellular tissue of the neck. There was a deep seated cellulitis involving the cellular tissue right down to the trachea.—*Jou. Brit. D. Asso.*

---

**EXTRACT FROM A DISCUSSION ON ANCHORAGE OF GOLD FILLINGS.**—Dr. Darby always felt a little sad when men whom he had learned to respect and hold in his professional esteem recommended such practice as to use sticking plasters to hold their fillings in place. It is not good mechanics, it is not good sense, and he did not believe it to be good practice. Old practitioners should use great care in advocating such things, in place of the more reasonable practice of making the cavities of a retentive form, and then thoroughly consolidating the material into them. We are led into bad practice by these means. Some years ago there was a craze for copper amalgam. Some of us were a little doubtful concerning it until Dr. Miller pronounced in favor of it, and then we thought we were entirely safe in following in the footsteps of so eminent a man. Now we can see that scarcely anything has done so much harm in dental practice as copper amalgam. A great many teeth and some dentists have been ruined by it.

Our young men are always looking for some patent method of easy practice, and some of our older practitioners keep their eyes peeled for short cuts of excellence. There is but one road to success, and that is patient, painstaking effort. There are more patent suckers for holding artificial plates in the mouth than a man can reckon up without taking a great deal of time to it, but who ever heard of a reputation for good work being won by means of them? If a plate is without adaptation, all the gimcrack stickers that were ever peddled out by men too lazy to earn a reputable living will not make it what it should be, and it is the same with fillings.—*Practitioner and Advertiser.*

---

ARTICULATING LOWER PARTIALS.—When articulating to the upper natural teeth, but little trouble is experienced. Be very careful and make the bearing heaviest on inside cusps of second bicuspids, and first molars; see that the plate is not displaced in closing the mouth. In articulating these partials to full upper sets, is where the trouble comes. I have made a good many sets for pocket pieces and dresser ornaments before I succeeded in doing the work with sure results. I now make the upper set first, complete it, fit in the mouth, and if for a home patient let them wear the plate till settled to place. Now take the impression for lower partial; make cast, and trial plate, try the plate in the mouth and trim to fit. Wax up with spatula and dry heat, building wax on where the teeth are to go, for the bite. Put the dry plate in the mouth and let the patient close the upper teeth into the wax; now remove altogether in this position, and mount in the articulator. Grind and fit on the teeth, try in; you will probably find the articulation all right, but if you notice the slightest displacement in closing the mouth, correct it before you go any farther. I have used this method for twelve years with the best satisfaction to myself and patients.—*Items of Interest.*

---

MAKING METAL DIES FROM PLASTER MOLDS.—Take the impression and make the plaster model as usual. When the plaster sets, trim and shape it as you want the metal die; cover the model with heavy tinfoil, making a perfectly smooth fit. Now lay the covered model on a piece of glass, tin side up, and cover it all over with plaster, from one-fourth to one inch thick, making it thick on palatine part, and thin on rim; especially if there is a good deal of undercut. When the plaster sets remove the model. If the rim breaks in separating, replace, and back up with new plaster mixed thin. This gives a tin-lined mold for casting in. Put the mold over the kerosine stove, and when hot and dry, pour in the zinc or Babbitt metal. The metal should not be too hot when poured. In putting the tin on the mold, and in seperating the model from it, care must be taken not to tear or puncture the tin. Smoke the die, or coat with whiting, and cast the counter as usual.

---

WELDING GOLD CROWNS.—Dr. G. W. Melotte describes a method of welding gold bands for use in crown- and bridge-work, producing a seamless band in which the line at which the ends were joined was no harder than any other part of the band, as it must be when the union is made with solder of lower carat than the band. The process is to bevel the edges to be joined with a short bevel, bringing the parts to perfect adaptation, then apply borax ground in water to the consistence of cream, and with the soft flame of a blow pipe bring the gold to the point of fusion. The molecules of the gold will unite, making a continuous seamless band.—*Items of Interest.*

DEATHS UNDER ANÆSTHETICS.—Gurlt (*Rathgeber für Gesunde und Kranke*, November 19, 1892,) reported to the last Surgical Congress at Berlin the following statistics of deaths under anæsthetics. They are made up from the observations of 62 operators, who anæsthetized 109,196 persons, with 39 fatal results, showing 1 death to 2,800 narcoses. The following were the anæsthetics used:

| | | |
|---|---|---|
| Chloroform . . . . . . . . | 94,123 narcoses, | 36 deaths. |
| Ether . . . . . . . . . . . . | 9,431 narcoses, | no deaths. |
| Ether and chloroform . . | 2,891 narcoses, | 1 death. |
| Ether and alcohol . . . . | 1,384 narcoses, | no deaths. |
| Bromoform with ethyl bromide . . . . . . . . . | 2,151 narcoses, | 1 death. |
| Penthal . . . . . . . . . . | 219 narcoses, | 1 death. |

In 2,913 cases the narcosis lasted over an hour; in an operation for utero-vaginal fistula, 4½ hours; in a case of tetanus, 9 hours. In 25 cases, of which post-mortem examinations were made, cardiac diseases were found. The author urges careful examination of the heart before administering chloroform.

---

WHAT TO DO IN CHLOROFORM SYNCOPE.—In his work on this subject, Dr. Bobroff says in the same journal:

1. Our whole course in chloroform syncope must be revised.

2. We must not be unsystematical in the selection of means.

3. Injections of ether and alcohol under the skin, as well as the inspiration of amylnitrate, are hurtful, and must be discarded.

4. The injection of a salt solution is a good and harmless remedy. Indeed, why bring all sorts of poisons into the system when the desired aim, the lifting of blood pressure, and the heart action, can be achieved by the use of an innocent liquid.

5. If the salt Injection has no effect, and respiration ceases with the heart action, it is necessary to resort immediately to artificial respiration.—*Items of Interest.*

